# The -308G/A of Tumor Necrosis Factor (TNF)-α and 825C/T of Guanidine Nucleotide Binding Protein 3 (GNB3) are Associated with the Onset of Acute Myocardial Infarction and Obesity in Taiwan

**DOI:** 10.3390/ijms13021846

**Published:** 2012-02-09

**Authors:** Wei-To Chang, Yi-Cheng Wang, Chun-Chang Chen, Shi-Kun Zhang, Chen-Hsun Liu, Fu-Hsin Chang, Li-Sung Hsu

**Affiliations:** 1 Division of Cardiology, Kaohsiung Armed Forces General Hospital, Kaohsiung 802, Taiwan; E-Mails: wtc911@ms36.hinet.net (W.-T.C); cvwang@ms25.hinet.net (Y.-C.W.); ks802.ccc@msa.hinet.net (C.-C.C); lwgrita20@gmail.com (S.-K.Z.); 2 Department of Biological Science and Technology of I-Shou University, Kaohsiung 840, Taiwan; E-Mail: caca0514@hotmail.com.tw; 3 Department of Biomedical Research, Asia-Pacific Biotech Developing, Kaohsiung 806, Taiwan; E-Mail: bio_apd@yahoo.com.tw; 4 Institute of Biomedical Sciences of National Sun Yat-Sen University, Kaohsiung 804, Taiwan; 5 Institute of Biochemistry and Biotechnology, Chung Shan Medical University, Taichung 402, Taiwan; 6 Clinical Laboratory, Chung Shan Medical University Hospital, Taichung 402, Taiwan

**Keywords:** acute myocardial infarction, gene polymorphisms, *GNB-3*, TNF-alpha

## Abstract

Acute myocardial infarction is a highly prevalent cardiovascular disease in Taiwan. Among several etiological risk factors, obesity and inflammation are strongly associated with the frequency of hypertension, cardiovascular disease, diabetes, and myocardial infarction. To discriminate obesity- and inflammation-related genes and the onset of acute myocardial infarction (AMI), a case-control study was conducted to investigate the association of the -308G/A polymorphisms of tumor necrosis factor (TNF)-α and the C825T polymorphism of guanidine nucleotide binding protein 3 (GNB3*)* with the onset of AMI among Taiwanese cohorts. A total of 103 AMI patients and 163 matched normal control samples were enrolled in the present study. The genomic DNA was extracted and subjected into polymerase chain reaction-based restriction fragment length polymorphism (PCR-RFLP) analysis. An association between the A homozygosity of the TNF-α-308G/A polymorphism and the onset of AMI was observed among the male subjects (*p* = 0.026; Spearman index = 0.200, *p* = 0.008). An association between the T homozygosity of *GNB3* C825T polymorphism and obesity was also observed (Fisher’s exact, *p* = 0.009). The TT genotype has a protective effect against acquiring AMI among the obese female population in Taiwan (Fisher’s exact, *p* = 0.032). In conclusion, TNF-α-308G/A and the *GNB3* C825T polymorphisms are associated with obesity and AMI in the Taiwanese population.

## 1. Introduction

Acute myocardial infarction (AMI) is one of the leading causes of death worldwide. AMI has a high prevalence in Taiwanese cohorts, especially the elderly [1]. However, the age of AMI onset has decreased because of environmental risk factors and genetic polymorphisms [2].

Tumor necrosis factor-α (TNF-α), a key pro-inflammatory factor, has been linked to several human diseases. The -308G/A polymorphism in the promoter region of TNF-α is associated with its expression and effects, such as in lipid metabolism, insulin resistance, and endothelial function in cardiovascular disease [3]. A higher frequency of carriers of the A allele is observed in patients with unstable angina. A more striking association between A allele carriage frequency and unstable angina is found in patients with a body mass index (BMI) ≤ 27 (*p* = 0.012; odds ratio = 3.0) [4]. The association between the A allele carriage frequency and the development of atherosclerosis, as well as the presence of family history of coronary heart disease is also observed [5]. Antonicelli *et al*. demonstrated the higher frequency of the TNF-α-308AA+AG genotype among ST-elevation AMI patients compared with the non-ST-elevation AMI patients and normal individuals [6].

G-proteins are essential partners of the seven transmembrane receptors for the activation or inhibition of intracellular signaling cascades. Most vasoactive or growth stimulating factors communicate via G proteins in virtually all cardiovascular tissues [7]. Recently, a C825T polymorphism has been described in exon 10 of the β3 subunit of G protein (GNB3) [8]. The T allele results in the expression of a splicing variant with a deletion of 41 amino acids, including one tryptophan–aspartic acid (WD) domain [9]. The splicing variant is active and the cellular response increases [9]. Emerging reports have demonstrated that the C825T polymorphism of GNB3 is associated with different human diseases, such as hypertension [10], cardiovascular diseases [10,11], and ischemic stroke [12] According to a previous study, the C825T polymorphism of *GNB3*, T-homozygosity was found to be significantly more common among female myocardial infarction (MI) fatalities than the female control group (24% *versus* 7%; Relative Risk 2.29). The investigators concluded that the C825T polymorphism may play a role in the development of MI, at least among females [13]. The association of the T allele of the *GNB3* C825T polymorphism with arterial hypertension has been confirmed in smaller cohorts [8,14,15]. Some divergent results have also been obtained [16,17]. Several studies have also shown that young 825T allele carriers are predisposed to obesity, and this association could be confirmed across different ethnicities, including young Germans, Chinese, and Black Africans [18]. In the present study, we aim to investigate the association between the -308G/A polymorphisms of TNF-α and the C825T polymorphism of *GNB3* in terms of the onset of AMI among Taiwanese cohorts.

## 2. Results

The genetic frequencies of the TNF-α-308G/A and the *GNB3* C825T polymorphisms among the different subjects are shown in Table 1. A significantly reduced frequency of the T homozygote of the *GNB3* C825T polymorphism was observed in the BMI ≥ 27 subgroup (*p* = 0.009; Spearman index = −0.381, *p* = 0.003; OR = 0.548, 95% CI = 0.331–0.910). However, no differences were observed in the genetic frequency distribution of the TNF-α-308G/A polymorphism when the AMI patients were compared with the normal subjects (group IIA).

The T allele carriage is beneficial for avoiding the onset of AMI among obese women in Taiwanese cohorts (Table 2, *p* = 0.032).

A high frequency of T allele carriage was also found among the AMI patients and the healthy control subjects. The T homozygosity acts as a predisposing factor for obesity (Table 3, χ^2^ = 7.656, *p* = 0.006; Spearman index = 0.19, *p* = 0.006; OR = 1.437, 95% CI = 1.135–1.818). The T homozygosity of the *GNB3* C825T polymorphism results in a transcriptional truncated variant, and through the signal-enhancing ability of the G protein, it modulates adipogenesis and obesity risk. Furthermore, it is also associated with essential hypertension. However, a correlation between the T homozygote and the onset of AMI was not observed among Taiwanese cohorts, although a protective effect was observed within the obese female population. The results still need further confirmation via large-scale gene-associated studies within the Taiwanese cohorts. The controversial results might provide other clues as to whether this polymorphism does not directly affect the onset of AMI whether in males or females. The results might also modulate the AMI risk through the unknown mechanism, and this characteristic might differ among different ethnic groups.

For the TNF-α-308G/A polymorphism, we referred to the analyses that the association of gender might have an influence upon the AMI onset in advance because obesity might not be involved. An association between the A allele homozygosity of TNF-α-308G/A polymorphism with AMI within the male population was confirmed, but not in the female cohorts (Table 4, χ^2^ = 4.948, *p* = 0.026; Spearman index = 0.200, *p* = 0.008). The G to A substitute of the -308 nucleotide located in the promoter region of the TNF-α gene would increase its mRNA transcription level and contribute to the elevation of serum TNF-α secretion. The increased pro-inflammatory cytokine may accelerate the formation of plaque rupture via the induction of unusual apoptosis activities of the cardiomyocytes, and modulates the AMI onset risk. Our finding was consistent with the current point of view. The male gender is considered a risk factor for the onset of AMI, but the exact genetic basis has not been investigated very well. The polymorphisms of inflammatory cytokines might probably be involved in this sex difference.

## 3. Materials and methods

### 3.1. Patients

Up to 103 (82 men, 21 women; mean age = 60; obese = 20, non-obese = 83) Taiwanese patients with AMI recently registered in the Cardiovascular Disease Study from January 2006 to September 2006 in the Cardiology Division of Kaohsiung Armed Forces General Hospital or old AMI cases since March 1998 were recruited upon their return to the out-patient service of Kaoshiung Military General Hospital for their regular physical exams. Obesity was defined as having a BMI ≥ 27. Another group of 163 (92 men, 71 women; mean age = 53; obese = 39, non-obese = 124) healthy individuals were recruited as the control subjects. Among the control subjects, 109 patients who have no history of AMI but have other cardiovascular symptoms were recruited as control group IIA (non-AMI subgroup), whereas 54 who have no conventional cardiovascular disease risk factors were designated as control group IIB (low conventional risk of CAD subgroup). The conventional risk factors were described in a previous report [19]. Informed consent was obtained from each person. The present study was approved by the International Review Board, and complied with the Guidelines for Genetic Research of Kaohsiung Armed Forces General Hospital.

### 3.2. Genotyping

**Figure 1:**
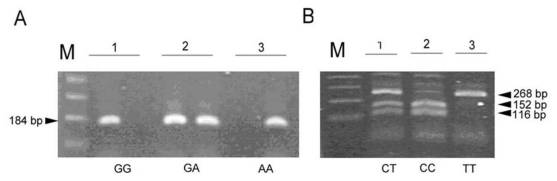
The polymorphism of TNF-α-308G/A and *GNB3* C825T. (**A**) the 184 bp genotype specific PCR products of TNF-α-308G/A polymorphism were amplified by using ARMS-PCR as described in Methods. Two independent PCR reactions for allele-specific amplification of each clinical sample were performed and separated by gel electrophoresis. The gel image revealed three typical genotypes of this polymorphism; (**B**) The patterns of *GNB3* C825T polymorphism after digested with BsajI restriction enzyme were shown. This pattern consisted of CT: 268 bp, 152 bp, and 116 bp digested fragments; CC: 152 bp and 116 bp digested fragments; TT: an indigested 268bp DNA fragment. M: 100 bp DNA maker.

**Figure 2:**
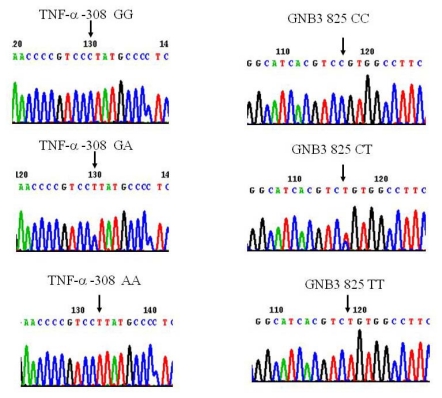
DNA sequence analysis of polymorphism of TNF-α and GNB3. Amplicons of TNF-α-308G/A (left panel) and GNB3 C825T were subjected into directed DNA sequence analysis using reverse (TNF-α-308G/A) and forward (GNB3 C825T) primers. The polymer sites were indicated with arrows.

Peripheral blood (4 mL) was collected from each subject into vacuum tubes containing 50 mmol/L ethylenediamine tetraacetic acid. Genomic DNA was isolated with a NucleoSpin^®^ Blood Genomic DNA kit (Macherey-Nagel, Duren, Germany). The A and G alleles at position -308 in the promoter region of the TNF-α gene were identified using the amplification refractory mutation system polymerase chain reaction (ARMS-PCR) methodology as previously described, with minor modifications [20]. A total of 100 ng of genomic DNA was used in each ARMS-PCR reaction. The PCR primer sequences were as follows: Generic primer, TNFA-R (antisense): 5’-tctcggtttcttctccatcg-3’, Primer specific for G allele, TNFA-F1 (sense): 5’-ataggttttgaggggcatgg-3’, Primer specific for A allele, TNFA-F2 (sense): 5’-aataggttttgaggggcatga-3’. The 20 μL basic PCR reaction mixture contained 100 ng of DNA, 10 pmol of each primer, 0.25 mmol/L of premixed deoxynucleoside triphosphate, 0.5 mmol/L, and 1 U YEA Taq polymerase (Yeastern Biotech, Taipei, Taiwan). The PCR conditions for the TNF-α-308G/A SNP amplification comprised two major thermal cycling procedures: an initial denaturation at 95 °C for 1 min; 10 cycles of denaturation at 95 °C for 15 s, annealing at 63 °C for 45 s, extension at 72 °C for 35 s; 25 cycles of denaturation at 95 °C for 20 s, annealing at 59 °C for 45 s, extension at 72 °C for 45 s, and a final extension at 72 °C for 5 min. The resulting amplified ARMS-PCR product was 184 bp (Figure 1A). The *GNB3* C825T polymorphism was determined by PCR-RFLP analysis. The primers used in the PCR were those described by Siffert *et al.* [9]. The PCR conditions comprised initial denaturation at 94 °C for 5 min; 35 cycles of denaturation at 94 °C for 15 s, annealing at 60 °C for 30 s, extension at 72 °C for 30 s, and a final extension at 72 °C for 8 min. The 268 bp PCR product was digested with *Bsa*JI for 2–3 h at 37 °C. Thus, the *Bsa*JI restriction site would be destroyed if the PCR product included a C to T substitution, whereas the C allele generated two fragments, 152 and 116 bp (Figure 1B). The amplified DNA or enzyme-restricted fragments were separated using 2% Amresco^®^ Agarose I™ agarose (Solon Ind., Solon, OH, USA), and then stained with 0.5 μg/mL ethidium bromide solution, and visualized by Stratagene Eagle Eyes^®^ II UV transilluminator (Stratagene, La Jolla, CA, USA). The gel image was recorded with a digital gel documentation system. To confirm the accuracy of genotyping by this method, we randomly selected 10% DNA samples and subjected them to direct DNA sequencing. In each instance, the genotype determined by the allele-specific PCR assay system or PCR-RFLP was identical to the samples determined by the confirmatory methods. The genotype of each polymorphism was confirmed by at least two well-trained laboratory technicians. To further confirm the PCR-RFLP results, directed DNA sequence analysis using forward and reverse primers was also performed (Figure 2).

### 3.3. Statistical Analysis

The experimental data on the gene polymorphisms were converted into categorical variables and compared with a χ^2^ test, Fisher’s exact test, and Spearman’s correlation test to distinguish the significance of the variation among the subjects. Allele frequencies were estimated through the gene-counting method. The χ^2^ test was used to identify departure from the Hardy–Weinberg equilibrium. Each polymorphism was assessed according to dominant, recessive, and additive genetic models. The odds ratio and 95% confidence interval of each genotype conferring to AMI risk were also calculated. The statistical significance was defined as having a *p* value lower than 0.05, and all statistical methods were performed using the commercial statistical package software SPSS 13.0 for Windows (SPSS, Inc., Chicago, IL, USA).

## 4. Discussion

AMI is a polygenic illness, and its molecular genetic levels have been studied for quite some time. Many susceptible gene candidates have been confirmed to be highly associated with the onset and prognosis of AMI [21,22]. Even some well-known etiologic risk factors probably have genetic backgrounds. The interaction between environmental risk factors and the susceptible gene polymorphisms is highly interesting. Hypertension, obesity, and cardiogenic inflammation are the common disorders that constitute a major risk factor for cardiovascular diseases, including kidney and cerebrovascular diseases [23]. Although the underlying molecular mechanisms remain largely elusive, recent advances in SNP studies have provided strong evidence that the risk of AMI is variable according to interpopulation or intrapopulation, and is largely determined or modulated by genetic factors.

Growing evidence shows that inflammation plays a central role in the pathogenesis of AMI [24]. Among the factors that promote inflammation and arterial thrombosis, one of the most important is the proinflammatory cytokine TNF-α [25]. The expression of this cytokine is modulated by a polymorphism located at nucleotide -308 of the TNF-α promoter gene [26]. There is a significant association between the TNF-α-308 polymorphism and the occurrence of ST-elevated myocardial infarction (STEMI), which suggests that this polymorphism plays a role in the pathogenesis of cardiac ischemic damage [27]. High plasma levels of biochemical ischemic markers have been found among AA+AG TNF-α-308 genotype carrier individuals, which shows that they would likely be affected by more severe ischemic damage than the rest of the population [6]. Furthermore, the homozygous A allele carriage of this polymorphism has been associated with unstable angina [4] and susceptibility to plaque formation, in accordance with its mechanisms. Based on these findings, A allele carriage is not associated with the risk of CAD or MI in the angiographically examined patients [28]. However, A allele carriage is commonly believed to predispose to CAD and MI. In the present study, an association between A homozygous allele carriage and the onset of AMI was observed among male Taiwanese subjects, which might be independent of obesity. Polymorphisms of proinflammatory cytokines, such as *IL-6* -174 G > C, have also been associated with acute coronary syndrome (ACS), resulting in higher risk of death among elderly ACS male patients [29]. Polymorphism of proinflammatory cytokines is more likely to be sex-dependent because of its contribution to the pathogenesis of AMI. This hypothesis still needs to be confirmed. According to previous TNF-α-308G/A SNP studies performed in Taiwanese cohorts, the A homozygous carriage is seldom detected, only ranging from 0.37%–1.25%, and it is not associated with hypertension and type II DM [30,31] (The lower frequency obtained in the present study was 4.13%, which might also reflect the nature of genetically predisposing factors.

For the genetic variant of the *GNB3* gene, Siffert *et al*. first described a C825T polymorphism in exon 10 of the *GNB3* gene, which encodes the β3-subunit of G-protein. The 825T allele was found to be associated with the expression of a truncated but functionally active splice variant of the β3-subunit, an enhanced intracellular signal transduction of G-protein cascade, and an increased risk of hypertension [9]. As a result, alterations, such as C to T substitution at nucleotide 825 that affect G-protein function or expression may have a strong influence on cellular signaling, consequently modulating a wide range of disorders [10,32]. However, arguable results were obtained; most studies in Caucasian populations support the idea that the 825T allele increases the risk for hypertension. In contrast, studies among Black and East Asian populations show more controversial results [10]. The highest frequencies of the 825T allele (up to 80%) are found among old ethnic groups, e.g., Black Africans, African Americans, Bushmen, and Australian aborigines. The enhanced G protein activation represents a thrifty genotype, which might have facilitated the survival of our ancestors. The frequencies of the 825T allele are significantly lower in Asians (approximately 40 to 50%) and Caucasians (30%). Excluding the influence on blood pressure control, many studies examined the potential association between *GNB3* C825T polymorphism and several other phenotypes of clinic traits, such as obesity [18,32,33], atherosclerosis [11,34], myocardial infarction [35], radial artery hypertrophy [36], and diabetes [37]. The widely accepted concept is that the *GNB3* 825T allele carriage results in hypertension. However, limited evidence of its clinical relevance to the onset of AMI has been identified. Some phenomena suggest that the *GNB3* 825T allele carriage might interact with the D allele of *ACE* I/D polymorphism, and probably has a combined effect on MI [35], or is associated with the onset of AMI among female Caucasians cohorts. These divisive observations have been mentioned in a previous study [38]. In addition, Hengstenberg *et al.* have shown that the prevalence of TT genotype did not show any significant difference in female individual in population-base survey compared to MI patients [38]. On the other hand, Klintschar *et al.* have demonstrated that T homozygous frequency was higher in female myocardial infarction group compared to normal control group, whereas no difference was found in male [39]. In this study, we observed that TT genotype has a protective effect against acquiring AMI among the obese female population in Taiwan. These differences may results from distinct ethnic groups. Interestingly, homozygous T allele carriage of the *GNB3* C825T was found to be quite common within Taiwanese population (50%–60%). The homozygous T allele carriage of the *GNB3* C825T is associated with obesity, but somehow has an opposite effect on the onset of AMI among our studied population.

## 5. Conclusions

Genotyping at the TNF-α and *GNB3* loci represents an ideal tool for preventive medicine, in that individuals at risk of obesity and AMI can be identified early and their genetic predisposition could be counteracted through changes in lifestyle. For individuals with borderline AMI, genotyping can facilitate decisions related to medical treatment as a positive test confirms an inherited form of AMI.

## Figures and Tables

**Table 1:** The allele frequency distribution of Tumor necrosis factor-α (TNF-α) -308G/A and guanidine nucleotide binding protein 3 (*GNB3)* C825T polymorphism.

TGF -308	G/A	*p* value	Spearman Colleration	O.R. (95%C.I.)
AA *vs.* GG+GA				
BMI >= 27	AMI/IIA			
	GG 65%/54%	0.063	0.323 (0.013)	ND
	GA 20%/46%			
	AA 15%/0%			
BMI < 27	GG 72%/69%	1.0	0.013 (0.087)	1.124 (0.260–4.855)
	GA 23%/27%			
	AA 5%/4%			
GNB C825T				
TT *vs.* CC+CT				
BMI >= 27	AMI/IIA			
	CC 10%/5%	0.009 **	−0.381 (0.003)	0.548 (0.331–0.910)
	CT 45%/13%			
	TT 45%/82%			
BMI < 27				
	CC 13%/21%	0.908	0.022 (0.783)	1.048 (0.753–1.048)
	CT 37%/31%			
	TT 50%/48%			

O.R.: odd ratio; ND: not determine;

** *p* < 0.001.

**Table 2:** The allele frequency distribution of *GNB3* C825T polymorphism for advanced analyses in relation to gender.

		*p* value	Spearman Correlation	O.R. (95%C.I.)
BMI >= 27, female				
	AMI/IIA			
TT	0%/89%	0.032 *	−0.667 (0.001)	ND
CC+CT	100%/11%			
BMI >= 27, male				
	AMI/IIA			
TT	50%/76%	0.108	−0.272 (0.094)	0.658 (0.390–1.104)
CC+CT	50%/24%			

O.R.: odd ratio, ND: not determine;

* *p* < 0.05.

**Table 3:** The allele frequency distribution of *GNB3* C825T polymorphism for advanced analyses in relation to obesity.

		*p* value	Spearman Correlation	O.R. (95%C.I.)
	BMI > 27/BMI < 27			
TT	70%/48%	0.006 **	0.19 (0.006) **	1.437 (1.135–1.181)
CT+CC	30%/52%			

O.R.: odd ratio;

** *p* < 0.001.

**Table 4:** The allele frequency distribution of TNF-α-308G/A polymorphism for advanced analyses in relation to gender.

		*p* value	Spearman Correlation	O.R. (95%C.I.)
AA *vs.* GG+GA				
Male	AMI/IIA+IIB			
AA	7%/100%	0.026 *	0.2 (0.008)	ND
GA+GG	93%/0%			
Female				
AA	5%/6%	1.00	−0.016 (0.879)	0.845 (0.100–7.159)
GA+GG	95%/94%			

O.R.: odd ratio, ND: not determine;

* *p* <0.05.

## References

[1] Wang Y.C., Hwang J.J., Hung C.S., Kao H.L., Chiang F.T., Tseng C.D. (2006). Outcome of primary percutaneous coronary intervention in octogenarians with acute myocardial infarction. J. Formos. Med. Assoc.

[2] Liu P.Y., Chen J.H., Li Y.H., Wu H.L., Shi G.Y. (2003). Synergistic effect of stromelysin-1 (matrix metallo-proteinase-3) promoter 5A/6A polymorphism with smoking on the onset of young acute myocardial infarction. Thromb. Haemost.

[3] Vendrell J., Fernandez-Real J.M., Gutierrez C., Zamora A., Simon I., Bardaji A., Ricart W., Richart C. (2003). A polymorphism in the promoter of the tumor necrosis factor-alpha gene (-308) is associated with coronary heart disease in type 2 diabetic patients. Atherosclerosis.

[4] Bernard V., Pillois X., Dubus I., Benchimol D., Labouyrie J.P., Couffinhal T., Coste P., Bonnet J. (2003). The -308 G/A tumor necrosis factor-alpha gene dimorphism: A risk factor for unstable angina. Clin. Chem. Lab. Med.

[5] Dedoussis G.V., Panagiotakos D.B., Vidra N.V., Louizou E., Chrysohoou C., Germanos A., Mantas Y., Tokmakidis S., Pitsavos C., Stefanadis C. (2005). Association between TNF-alpha -308G>A polymorphism and the development of acute coronary syndromes in Greek subjects: The CARDIO2000-GENE Study. Genet. Med.

[6] Antonicelli R., Olivieri F., Cavallone L., Spazzafumo L., Bonafe M., Marchegiani F., Cardelli M., Galeazzi R., Giovagnetti S., Perna G.P. (2005). Tumor necrosis factor-alpha gene -308G>A polymorphism is associated with ST-elevation myocardial infarction and with high plasma levels of biochemical ischemia markers. Coron. Artery Dis.

[7] Kang M., Chung K.Y., Walker J.W. (2007). G-protein coupled receptor signaling in myocardium: not for the faint of heart. Physiology (Bethesda).

[8] Schunkert H., Hense H.W., Doring A., Riegger G.A., Siffert W. (1998). Association between a polymorphism in the G protein beta3 subunit gene and lower renin and elevated diastolic blood pressure levels. Hypertension.

[9] Siffert W., Rosskopf D., Siffert G., Busch S., Moritz A., Erbel R., Sharma A.M., Ritz E., Wichmann H.E., Jakobs K.H. (1998). Association of a human G-protein beta3 subunit variant with hypertension. Nat. Genet.

[10] Siffert W. (2005). G protein polymorphisms in hypertension, atherosclerosis, and diabetes. Annu. Rev. Med.

[11] Wascher T.C., Paulweber B., Malaimare L., Stadlmayr A., Iglseder B., Schmoelzer I., Renner W. (2003). Associations of a human G protein beta3 subunit dimorphism with insulin resistance and carotid atherosclerosis. Stroke.

[12] Zhang L., Zhang H., Sun K., Song Y., Hui R., Huang X. (2005). The 825C/T polymorphism of G-protein beta3 subunit gene and risk of ischaemic stroke. J. Hum. Hypertens.

[13] Klintschar M., Stiller D., Schwaiger P., Kleiber M. (2005). DNA polymorphisms in the tyrosine hydroxylase and GNB3 genes: Association with unexpected death from acute myocardial infarction and increased heart weight. Forensic. Sci. Int.

[14] Beige J., Hohenbleicher H., Distler A., Sharma A.M. (1999). G-Protein beta3 subunit C825T variant and ambulatory blood pressure in essential hypertension. Hypertension.

[15] Dong Y., Zhu H., Sagnella G.A., Carter N.D., Cook D.G., Cappuccio F.P. (1999). Association between the C825T polymorphism of the G protein beta3-subunit gene and hypertension in blacks. Hypertension.

[16] Huang X., Ju Z., Song Y., Zhang H., Sun K., Lu H., Yang Z., Jose P.A., Zhou G., Wang M. (2003). Lack of association between the G protein beta3 subunit gene and essential hypertension in Chinese: a case-control and a family-based study. J. Mol. Med. (Berl.).

[17] Li B., Ge D., Wang Y., Zhao W., Zhou X., Gu D., Chen R. (2005). G protein beta 3 subunit gene variants and essential hypertension in the northern Chinese Han population. Ann. Hum. Genet.

[18] Siffert W., Forster P., Jockel K.H., Mvere D.A., Brinkmann B., Naber C., Crookes R., Du P.H.A., Epplen J.T., Fridey J. (1999). Worldwide ethnic distribution of the G protein beta3 subunit 825T allele and its association with obesity in Caucasian, Chinese, and Black African individuals. J. Am. Soc. Nephrol.

[19] Wang Y.C., Chen C.C., Zhang W.D., Zhang S.K., Chang F.H., Hsu L.S. (2010). The 252A/G and 804C/A polymorphisms of *Lymphotoxin-alpha* is associated to onset of acute myocardial infarction in Taiwan. LabMedicine.

[20] Govan V.A., Constant D., Hoffman M., Williamson A.L. (2006). The allelic distribution of -308 tumor necrosis factor-alpha gene polymorphism in South African women with cervical cancer and control women. BMC Cancer.

[21] Cambien F., Poirier O., Mallet C., Tiret L. (1997). Coronary heart disease and genetics in epidemiologist’s view. Mol. Med. Today.

[22] Incalcaterra E., Hoffmann E., Averna M.R., Caimi G. (2004). Genetic risk factors in myocardial infarction at young age. Minerva Cardioangiol.

[23] Ritchie S.A., Connell J.M. (2007). The link between abdominal obesity, metabolic syndrome and cardiovascular disease. Nutr. Metab. Cardiovasc. Dis.

[24] Elkind M.S. (2006). Inflammation, atherosclerosis, and stroke. Neurologist.

[25] Chen D., Assad-Kottner C., Orrego C., Torre-Amione G. (2008). Cytokines and acute heart failure. Crit. Care Med.

[26] Abraham L.J., Kroeger K.M. (1999). Impact of the -308 TNF promoter polymorphism on the transcriptional regulation of the TNF gene: relevance to disease. J. Leukoc. Biol.

[27] Padovani J.C., Pazin-Filho A., Simoes M.V., Marin-Neto J.A., Zago M.A., Franco R.F. (2000). Gene polymorphisms in the TNF locus and the risk of myocardial infarction. Thromb. Res.

[28] Koch W., Kastrati A., Bottiger C., Mehilli J., von Beckerath N., Schomig A. (2001). Interleukin-10 and tumor necrosis factor gene polymorphisms and risk of coronary artery disease and myocardial infarction. Atherosclerosis.

[29] Antonicelli R., Olivieri F., Bonafe M., Cavallone L., Spazzafumo L., Marchegiani F., Cardelli M., Recanatini A., Testarmata P., Boemi M. (2005). The interleukin-6 -174 G > C promoter polymorphism is associated with a higher risk of death after an acute coronary syndrome in male elderly patients. Int. J. Cardiol.

[30] Sheu W.H., Lee W.J., Lin L.Y., Chang R.L., Chen Y.T. (2001). Tumor necrosis factor alpha -238 and -308 polymorphisms do not associate with insulin resistance in hypertensive subjects. Metabolism.

[31] Shiau M.Y., Wu C.Y., Huang C.N., Hu S.W., Lin S.J., Chang Y.H. (2003). TNF-alpha polymorphisms and type 2 diabetes mellitus in Taiwanese patients. Tissue Antigens.

[32] Benjafield A.V., Lin R.C., Dalziel B., Gosby A.K., Caterson I.D., Morris B.J. (2001). G-protein beta3 subunit gene splice variant in obesity and overweight. Int. J. Obes. Relat. Metab. Disord.

[33] Stefan N., Stumvoll M., Machicao F., Koch M., Haring H.U., Fritsche A. (2004). C825T polymorphism of the G protein beta3 subunit is associated with obesity but not with insulin sensitivity. Obes. Res.

[34] Yamamoto M., Abe M., Jin J.J., Wu Z., Tabara Y., Mogi M., Kohara K., Miki T., Nakura J. (2004). Association of GNB3 gene with pulse pressure and clustering of risk factors for cardiovascular disease in Japanese. Biochem. Biophys. Res. Commun.

[35] Naber C.K., Husing J., Wolfhard U., Erbel R., Siffert W. (2000). Interaction of the ACE D allele and the GNB3 825T allele in myocardial infarction. Hypertension.

[36] Hanon O., Luong V., Mourad J.J., Bortolotto L.A., Safar M., Girerd X. (2002). Association between the G protein beta3 subunit 825t allele and radial artery hypertrophy. J. Vasc. Res.

[37] Kiani J.G., Saeed M., Parvez S.H., Frossard P.M. (2005). Association of G-protein beta-3 subunit gene (GNB3) T825 allele with Type II diabetes. Neuro. Endocrinol. Lett.

[38] Hengstenberg C., Schunkert H., Mayer B., Doring A., Lowel H., Hense H.W., Fischer M., Riegger G.A., Holmer S.R. (2001). Association between a polymorphism in the G protein beta3 subunit gene (GNB3) with arterial hypertension but not with myocardial infarction. Cardiovasc. Res.

[39] Klintschar M., Stiller D., Schwaiger P., Kleiber M. (2005). DNA polymorphisms in the tyrosine hydroxylase and GNB3 genes: Association with unexpected death from acute myocardial infarction and increased heart weight. Forensic. Sci. Int.

